# Breastfeeding and the risk of respiratory tract infections after infancy: The Generation R Study

**DOI:** 10.1371/journal.pone.0172763

**Published:** 2017-02-23

**Authors:** Ilse Tromp, Jessica Kiefte-de Jong, Hein Raat, Vincent Jaddoe, Oscar Franco, Albert Hofman, Johan de Jongste, Henriëtte Moll

**Affiliations:** 1 The Generation R Study Group, Erasmus MC, University Medical Center, Rotterdam, The Netherlands; 2 The Department of Pediatrics, Sophia Children’s Hospital, Erasmus MC, University Medical Center, Rotterdam, The Netherlands; 3 The Department of Epidemiology, Erasmus MC, University Medical Center, Rotterdam, The Netherlands; 4 The Department of Global Public Health, Leiden University College, The Hague, The Netherlands; 5 The Department of Public Health, Erasmus MC, University Medical Center, Rotterdam, The Netherlands; University of Tennessee Health Science Center, UNITED STATES

## Abstract

**Background:**

The protection of breastfeeding against respiratory tract infections in the first year of life has often been suggested. Few studies examined the effect of breastfeeding on respiratory tract infections after infancy.

**Objective:**

To examine the association between breastfeeding with lower respiratory tract infections (LRTI) and upper respiratory tract infections (URTI) after infancy up to 4 years of age (n = 5322).

**Methods:**

This study was embedded in The Generation R study, a Dutch population-based prospective cohort study from fetal life until young adulthood. Information on breastfeeding duration (never; <3 months; 3–6 months; ≥6 months) and dose (never; partially until 4 months; predominantly until 4 months) were collected by questionnaire at 2, 6, and 12 months of age. Information on doctor attendance for LRTI and URTI were obtained by questionnaire at 2, 3, and 4 years of age.

**Results:**

Breastfeeding for 6 months or longer was significantly associated with a reduced risk of LRTI up to 4 years of age (aOR: 0.71; 95% CI: 0.51–0.98). Similar ORs for LRTI were found with breastfeeding for less than 3 months and 3–6 months. Although in the same direction, weaker ORs were found for URTI and breastfeeding duration. The same trend was found for partial and predominant breastfeeding until 4 months and LRTI and URTI.

**Conclusion:**

Breastfeeding duration for 6 months or longer is associated with a reduced risk of LRTI in pre-school children. These findings are compatible with the hypothesis that the protective effect of breastfeeding for respiratory tract infections persist after infancy therefore supporting current recommendations for breastfeeding for at least 6 months.

## Introduction

Infectious diseases, including respiratory tract infections, are a leading cause of morbidity and hospitalization in infants and children.[1, 2] There is much epidemiological evidence for the benefits of breastfeeding against a wide range of infections and illnesses.[3, 4] Breast milk contains various antimicrobial substances, anti-inflammatory components and factors that promote immune development.[4, 5] It enhances the immature immune system of the infant and strengthens defense mechanisms against infectious and other agents during the breastfeeding period.[4–7] Exclusive breastfeeding for the first 6 months of life with breastfeeding along with complementary feeding thereafter is recommended by the World Health Organization (WHO).[8] The benefits have been found to be dose-dependent and related to the duration of breastfeeding.[3, 4] The protection of exclusive and prolonged breastfeeding against respiratory tract infections in the first year of life has often been suggested and also found in The Generation R Study.[3, 4, 9, 10] But, not all studies found breastfeeding exclusivity and duration to reduce the occurrence of respiratory tract infections.[11, 12] It is suggested that the influences of breast milk on the infant’s immune system may persist beyond the breastfeeding period, as it not only provides passive immunity but also maturation of the immune system in the long run.[13, 14] Since breastfeeding might protect against diseases in adulthood such as type 1 diabetes and inflammatory bowel disease [3, 4, 15] a prolonged protection against respiratory tract infections after the first year of life seems plausible. However, only few studies have examined the effect of breastfeeding on respiratory tract infections after infancy and reported inconsistent results.[16–21]

The aim of this study was to examine the association between breastfeeding and lower and upper respiratory tract infections after infancy up to 4 years of age.

## Subjects and methods

### Participants and study design

This study was embedded in the Generation R study, a population-based prospective cohort study from fetal life until young adulthood and has been described in detail previously.[22] In total, 9778 mothers with a delivery date from April 2002 through January 2006 enrolled in the study. Consent for postnatal follow-up was provided by 7893 participants (S1 Fig). The study was approved by the medical ethical review board of the Erasmus Medical Center, Rotterdam, the Netherlands.

### Respiratory tract infections

Data on respiratory tract infections was obtained by postal parent-reported questionnaires at the age of 2, 3 and 4 years. Parents were asked whether their child had suffered from a respiratory tract infection in the previous year and had visited a doctor for the infectious disease. Information on upper respiratory tract infections was obtained by asking parents whether their child had suffered from a serious cold, ear infection or throat infection. Information on lower respiratory tract infections was obtained by asking parents whether their child had pneumonia or bronchitis. Upper and lower respiratory tract infections were defined as present or absent in the second, third and fourth year of life. Questionnaire response rates were 76%, 69% and 73% at the age of 2, 3 and 4 years respectively. No information was available for the number of episodes or severity of these infections.

### Breastfeeding

Data on breastfeeding were collected by a combination of delivery reports and postnatal postal questionnaires at the age of 2, 6 and 12 months. By questionnaire the mothers were asked whether they had ever breastfed their child and at which age (in months) the child had stopped receiving breast milk. The duration of breastfeeding was categorized as (I) never breastfed, (II) breastfed for less than 3 months, (III) breastfed for 3–6 months, and (IV) breastfed for 6 months and longer. The majority of infants had stopped receiving breast milk before the age of 12 months, only 3 infants thereafter. An approximation of breastfeeding exclusivity was defined on the basis of parent reports of the child’s age at which solid foods were first introduced, including the introduction of formula feeding. Predominant breastfeeding was defined as receiving breastfeeding without any other infant formula, milk or solid foods.[23] Breastfeeding dose was categorized as (I) never breastfed, (II) partially breastfed until 4 months, and (III) predominantly breastfed until 4 months. Partial breastfeeding was defined as receiving both breast milk and infant formula and/or solid foods. Questionnaire response rates were 82%, 73% and 72% at the age 2, 6 and 12 months respectively.

### Covariates

Information on potential confounders, including mode of delivery, gender and gestational age, were obtained from obstetric records assessed in mid-wife practices and hospital registries.[22] Additional information was obtained by a combination of prenatal and postnatal questionnaires completed by both parents. The questionnaires included information on maternal age, maternal educational level, maternal marital status, maternal ethnicity, household income per month, maternal BMI before pregnancy, any maternal smoking during pregnancy, any maternal alcohol use during pregnancy, parity and parental history of asthma or atopy. Ethnicity of the mother was defined as follows: if both parents were born in The Netherlands, the ethnicity of the mother was defined as Dutch; if one of the parents was born in another country than The Netherlands, that country applied; if parents were born in different countries other than The Netherlands, the country of the mother applied.[24] Ethnicity of the mother was categorized into Western (Dutch, European, American-Western, Asian-Western) and non-Western (American non-Western, Asian non Western, African, Turkish, Cape Verdean, Moroccan, Dutch Antillean, Surinamese, Oceania, and Indonesian). Maternal educational level was defined as follows; low: no education, primary school or less than 3 years of secondary school, mid: more than 3 years of secondary school, higher vocational training or bachelor's degree, and high: academic education.[25] Household income per month was categorized into two income-groups using the approximate monthly general labour income during the inclusion period of this study as cut off point (≤ € 2200 and > € 2200).[26] Postnatal questionnaire completed by the mother at the child’s age of 6 months included information on smoke exposure of the child inside and outside the home. Environmental smoking was defined as maternal smoking, smoking of mother or anybody else at home in the presence of the child and smoking in any other places in the presence of the child at the age of 6 months. Postnatal questionnaires completed by the mother at age 12 months included information on vitamin D supplementation in the previous 6 months and questionnaires at age 12 and 24 months included information on day-care attendance.

### Population for analyses

Children whose parents did not provide informed consent for the use of postnatal questionnaire data (n = 1885) and children without information on respiratory tract infections at ages 2 to 4 years (n = 2015) were excluded from the analysis. To prevent clustering, only one child per family within the Generation R cohort was included by random selection (n = 556 excluded). To reduce attrition bias, variables with missing values were multiple imputed (20 imputations) based on the correlation between the variable with missing values with other maternal and child characteristics (S1 Table).[27] Consequently data of 5322 children were available after multiple imputation for statistical analyses (S1 Fig).

### Statistical methods

First, independent Student’s t test and chi-square test were performed to test for differences in characteristics between the 4 groups of breastfeeding duration. Second, logistic regression analyses by using generalized estimating equations (GEE) were performed. Regression analysis by GEE assesses the association between two variables by correction for the within subject's dependence as a result of the repeated observations on lower and upper respiratory tract infections (age 2, 3 and 4 years) since repeated measurements within one individual are frequently correlated.[28] An unstructured working correlation structure was used in the GEE analyses as adjustment for the dependency between the repeated measurements, since the within-subject correlation coefficient for lower and upper respiratory tract infections between the three time points were different (r = 0.13–0.32). Logistic regression analysis with GEE was performed with lower respiratory tract infections and upper respiratory tract infections as dependent variables and breastfeeding as independent variable. All analyses were adjusted for the age (time) at which observations of illness were assessed to account for potential confounding by age as well as clustering of repeated measurement. The selection of potential confounders was performed by the alteration in odds ratio (OR) and kept in the multivariable model in case of an alteration of ≥ 10% in OR.[29] The pooled results of the 20 imputed datasets were reported in this paper as odds ratio’s (OR’s) and 95% confidence intervals (95% CIs). A *P*-value < 0 .05 was considered as statistically significant. The statistical analyses were carried out by using SPSS 22.0 for Windows (SPSS Inc, Chicago, IL).

## Results

### Study population

Maternal and child characteristics are presented in Table 1 and S1 Table. Out of 5322 children, 14% had suffered from at least one episode of lower respiratory tract infection in the second year of life, 8% in the third year and 6% in the fourth year of life (Table 2). At least one episode of upper respiratory tract infection was reported for 44% of children in the second year of life, 36% in the third year and 31% in the fourth year of life (Table 2).

**Table 1:** Maternal and child characteristics (n = 5322).

		Breastfeeding duration^¶^							
Characteristics		Never		< 3 months		3–6 months		≥ 6 months	
n = 5322		n = 893 (17%)		n = 1602 (30%)		n = 1093 (21%)		n = 1734 (32%)	
		Number—% or mean ± SD		Number—% or mean ± SD		Number—% or mean ± SD		Number—% or mean ± SD	
**Maternal characteristics**									
Maternal age—Mean (SD)		*		*					
		30.7	5.1	30.3	4.9	31.5	4.6	31.8	4.8
Educational level – n (%)		*		*					
	Low	230	26	384	24	129	12	262	15
	Mid	496	55	881	55	566	52	856	49
	High	167	19	337	21	398	36	616	36
Ethnicity – n (%)		*							
	Western	623	70	1057	66	786	72	1186	68
	Non-Western	270	30	545	34	307	28	548	32
Household income per month – n (%)						*			
	≤ 2200 euro	385	43	648	40	332	30	710	41
	>2200 euro	508	57	954	60	761	70	1024	59
Marital status – n (%)				*					
	No partner	105	12	185	12	88	8	157	9
	Married/ Living together	788	88	1417	88	1005	92	1577	91
Maternal BMI before pregnancy (kg/m^2^)—mean (SD)		*		*					
		23.9	4.3	23.9	4.4	23.1	3.6	23.1	3.6
Child exposure to smoke – n (%)		*		*		*			
	Never	471	53	929	58	699	64	1224	70
	Prenatal smoking, no environmental smoking	51	6	115	7	77	7	113	7
	Prenatal smoking and environmental smoking	198	22	340	21	154	14	160	9
	Environmental smoking, no prenatal smoking	173	19	218	14	163	15	237	14
Alcohol use during pregnancy – n (%)		*				*			
	Never	454	51	724	45	356	33	741	43
	Drank alcohol during pregnancy	439	49	878	55	737	67	993	57
Parental history of asthma or atopy – n (%)				*		*			
	No	462	52	833	52	581	53	840	48
	Yes	432	48	769	48	512	47	894	52
Parity – n (%)		*		*		*			
	0	468	52	1037	65	677	62	947	55
	≥1	426	48	565	35	416	38	787	45
Caesarean section – n (%)		*		*		*			
	No	742	83	1357	85	943	86	1547	89
	Yes	152	17	245	15	150	14	186	11
**Child characteristics**									
Male – n (%)									
		464	52	818	51	554	51	832	48
Gestational age at birth – n (%)		*		*		*			
	<37 weeks	52	6	88	5	61	6	72	4
	≥37 weeks	842	94	1514	95	1032	94	1662	96
Vitamin D supplementation age 6–12 months – n (%)				*		*			
	No	450	50	941	59	657	60	786	45
	Yes	444	50	661	41	436	40	948	55
Day care attendance first 2 years – n (%)				*		*			
	No	248	28	369	23	213	19	423	24
	Yes	646	72	1233	77	880	81	1311	76

* Significantly different from breastfeeding ≥ 6 months, *P* < 0.05

^¶^ Missing data for breastfeeding duration before multiple imputation n: 1083 (20%)

**Table 2:** Prevalence of lower and upper respiratory tract infections (n = 5322).

Outcome	Age 2				Age 3				Age 4			
	Original data		Multiple imputed		Original data		Multiple imputed		Original data		Multiple imputed	
	n	%	n	%	n	%	n	%	n	%	n	%
**LRTI**												
No	4019	87	4591	86	3952	93	4884	92	4071	95	5011	94
Yes	594	13	732	14	316	7	438	8	201	5	311	6
*Missing*	709	13			1054	20			1050	20		
**URTI**												
No	2598	57	2954	56	2788	66	3412	64	3073	72	3666	69
Yes	1970	43	2368	44	1442	34	1910	36	1211	28	1656	31
*Missing*	754	14			1092	21			1038	20		

### Duration of breastfeeding and respiratory tract infections

Compared to children who were never breastfed, breastfeeding for 6 months or longer was significantly associated with a decreased risk of lower respiratory tract infections after infancy up to 4 years of age (aOR: 0.71; 95% CI: 0.51–0.98) (Table 3) (S2 Table). Similar ORs for lower respiratory tract infections were found with breastfeeding for less than 3 months and breastfeeding for 3–6 months but this was not statistically significant (aOR: 0.75; 95% CI: 0.56–1.00 and aOR: 0.78; 95% CI: 0.53–1.13) (Table 3) (S2 Table). Although in the same direction, weaker ORs were found for upper respiratory tract infections and breastfeeding for less than 3 months, 3–6 months or 6 months and longer after adjustment for confounding variables (aOR: 0.86; 95% CI: 0.70–1.04 for less than 3 months, aOR: 0.91; 95% CI: 0.73–1.12 for 3–6 months and aOR: 0.85; 95% CI: 0.69–1.05 for 6 months and longer) (Table 3) (S2 Table). The effects of the duration of breastfeeding on respiratory tract infections did not differ between the ages of 2, 3 and 4 years (p_interaction_ >0.23 for lower and upper respiratory tract infections).

**Table 3:** Association between breastfeeding duration and lower and upper respiratory tract infections up to age 4 years (n = 5322).

		Lower respiratory tract infections		Upper respiratory tract infections	
Breastfeeding		Univariate model	Multivariable model 1	Univariate model	Multivariable model 1
	n (%)	OR (95% CI)	aOR (95% CI) ^a^	OR (95% CI)	aOR (95% CI) ^a^
Never	893 (17%)	*Reference*	*Reference*	*Reference*	*Reference*
< 3 months	1602 (30%)	0.75 (0.56–1.02)	0.75 (0.56–1.00)	0.87 (0.71–1.06)	0.86 (0.70–1.04)
3–6 months	1093 (21%)	0.72 (0.48–1.06)	0.78 (0.53–1.13)	0.80 (0.64–1.00)	0.91 (0.73–1.12)
≥ 6 months	1734 (32%)	**0.63 (0.46–0.87)**	**0.71 (0.51–0.98)**	**0.78 (0.62–0.98)**	0.85 (0.69–1.05)

OR: Odds Ratio; 95% confidence interval. OR’s are compared to never-breastfed.

^a^ Adjusted for caesarean section, maternal age, marital status, maternal ethnicity, maternal educational level, household income per month, maternal BMI before pregnancy, smoke exposure child, alcohol use during pregnancy, gender child, vitamin D supplementation age 6–12 months, day-care attendance in the first two years of life, gestational age at birth, parity and parental history of asthma or atopy.

### Dose of breastfeeding and respiratory tract infections

Partial breastfeeding until 4 months was significantly associated with a decreased risk of lower respiratory tract infections after infancy up to age 4 years (OR: 0.76; 95% CI: 0.59–0.99). However, the association did not remain significant after adjustment for confounders (aOR: 0.78; 95% CI: 0.59–1.02) (Table 4) (S3 Table). The same trend was found for predominant breastfeeding but not statistically significant (Table 4). Before multiple imputation, predominant breastfeeding was associated with lower respiratory tract infections (S3 Table). Although partial breastfeeding until 4 months and predominant breastfeeding until 4 months was not significantly associated with upper respiratory tract infections, the effect estimates were found to be in the same direction (aOR: 0.89; 95% CI: 0.72–1.10 and aOR: 0.93; 95% CI: 0.72–1.20) (Table 4) (S3 Table). The effects of breastfeeding dose on respiratory tract infections did not differ between the ages of 2, 3 and 4 years (pinteraction >0.59 for upper and lower respiratory tract infections).

**Table 4:** Association between exclusive breastfeeding and lower and upper respiratory tract infections up to age 4 years (n = 5322).

		Lower respiratory tract infections		Upper respiratory tract infections	
Breastfeeding		Univariate model	Multivariable model 1	Univariate model	Multivariable model 1
	n (%)	OR (95% CI)	aOR (95% CI) ^a^	OR (95% CI)	aOR (95% CI) ^a^
Never	862 (16%)	*Reference*	*Reference*	*Reference*	*Reference*
Partially until 4 months	2870(54%)	**0.76 (0.59–0.99)**	0.78 (0.59–1.02)	0.87 (0.72–1.05)	0.89 (0.72–1.10)
Predominantly until 4 months	1590 (30%)	0.66 (0.44–1.00)	0.72 (0.48–1.09)	0.84 (0.65–1.08)	0.93 (0.72–1.20)

OR: Odds Ratio; 95% confidence interval. OR’s are compared to never-breastfed.

^a^ Adjusted for caesarean section, maternal age, marital status, maternal ethnicity, maternal educational level, household income per month, maternal BMI before pregnancy, smoke exposure child, alcohol use during pregnancy, gender child, vitamin D supplementation age 6–12 months, day-care attendance in the first two years of life, gestational age at birth, parity and parental history of asthma or atopy.

## Discussion

In this population-based prospective birth cohort study we found children who were breastfed for 6 months or longer to have a reduced risk of lower respiratory tract infections after infancy. For breastfeeding for less than 3 months and 3–6 months similar direction of the effect estimates were found. Also, similar direction of the effect estimates were found for the association between the duration and dose of breastfeeding and upper respiratory tract infections but not significant.

Various studies, including a previous study within our cohort, found exclusive breastfeeding for 6 months to be protective for the development of respiratory tract infections in infancy, thereby supporting the recommendation of the WHO.[10, 19, 30] Our study found breastfeeding for 6 months or longer to be associated with a reduced risk for lower respiratory tract infections after infancy till the age of 4 years. Contrary to our findings, a prospective longitudinal study found that breastfeeding duration, including breastfeeding longer than 6 months, was not associated with pneumonia or lung infection in 6 year old children.[17] However, the association was only examined in children who initiated breastfeeding whereas we also included children who were never breastfed. In agreement with our findings on breastfeeding dose, Li et al [17] did not find breastfeeding exclusivity to be associated with lower respiratory tract infections. As for upper respiratory tract infections, we did not observe a significant association among children who were breastfed (duration and dose) compared to those who were never breastfed. Similarly, Chantry et al [19] found full breastfeeding for less than 6 months not to be associated with an increased risk of recurrent upper respiratory tract infections and recurrent otitis media in children 6–72 months of age. Li et al [17] also found no association between breastfeeding, including duration and exclusivity, and colds or upper respiratory tract infections among 6 year old children. The possibility that the protective effect of breastfeeding might wear off after breastfeeding cessation has previously been suggested.[31–33] Other studies that did find breastfeeding to be associated with a reduced risk of upper respiratory tract infections after infancy mainly focused on otitis media and mostly before the age of 3 years.[20, 34, 35] Some studies examined the effect of breastfeeding on respiratory tract infections in general. A Japanese study reported breastfeeding duration for 6–7 months to be borderline significantly associated with a reduced risk of hospitalization for respiratory tract infections between the age of 18–30 months.[21] Respiratory tract infections for which hospitalization is needed are often more serious and mainly infections of the lower respiratory tract [2, 36] which might explain the discrepancy between these latter results and those from our study. Conversely, another study did not find a protective effect of breastfeeding for all acute respiratory illness in children 1–6 years [20] which might be due to an overrepresentation of upper respiratory tract infections since these symptoms are more common in childhood.[2] The WHO definition of exclusive breastfeeding allows for ORS, drops and syrups but no other food or drink, not even water.[23] Therefore, this study examined the effect of predominant breastfeeding defined as no infant formula, milk or solid foods. This study cannot examine the effect of predominant breastfeeding per month neither for the duration of 6 months or longer due to small group size. The majority of mothers in the Netherlands do not continue breastfeeding after the age of 4 months.[37] Thus, our study precludes conclusions on the effect of exclusive breastfeeding for 6 months as defined by the WHO. In line with our findings, a birth cohort study from Hong Kong did not find exclusive and partial breastfeeding for 3 months to be associated with a reduced risk for hospital admissions for respiratory tract infections after the age of 6 months up to age 8 years.[16] However, Yamakawa et al.[21] did find exclusive breastfeeding at 6–7 months of age to be significantly associated with hospitalization for respiratory tract infections between the age of 18–42 months. Also, Li et al.[17] reported exclusive breastfeeding for 6 months and longer, compared to breastfeeding between 0 to 4 months, to be significantly associated with a reduced odds for ear, throat, and sinus infection at age 6 years.

We performed multiple imputation to account for bias associated with missing data. Children with and without questionnaire data differed in socioeconomic background, ethnicity, and a selection towards a relative more healthy study population seems to be present.[22] However, this would only affect the interpretation of our results if the association between breastfeeding and respiratory infections was different for children without questionnaire data compared with those with questionnaire data, which is unlikely. For the analyses on breastfeeding duration ≥6 months and lower respiratory tract infections, results were comparable in the original data (aOR: 0.56; 95% CI: 0.32–0.99) and after the multiple imputation procedure (aOR: 0.71; 95% CI: 0.51–0.98). However, for the analyses on predominant breastfeeding and lower respiratory tract infections the estimate in the original data analysis (aOR: 0.53; 95% CI: 0.30–0.93) slightly weakened after the imputation procedure (aOR: 0.72; 95% CI: 0.48–1.09). This would suggest that the missing data was not completely random and affected the uncertainty of the effect estimates for predominant breastfeeding.

An important strength of this study is the large study population drawn from the general population. On the basis of previous findings in our cohort, respiratory illnesses are socially patterned and related to several mother and child characteristics.[38] Our study design provided information on multiple potential confounders and allowed for follow-up into childhood. However, due to the observational nature of our study, residual confounding cannot be fully excluded. In addition, the prospective design made it possible to obtain information on breastfeeding at multiple time points during infancy therefore limiting recall bias. Whereas other studies examined the effect of respiratory tract infections in general, or focused on specific infections,[20, 21, 34, 35] we examined the effect of breastfeeding on the development of lower and upper respiratory tract infections separately.

A weakness may be that the diagnosis of respiratory tract infection was obtained by parent-reported questionnaires at yearly intervals. The questions used to obtain information on respiratory infections were comparable to other studies. Parents were asked whether their child had suffered from a respiratory tract infection and whether they had visited a doctor for this infection since physician diagnosis is more accurate. However, this could have led to misclassification of the outcome as parents may not be able to distinguish between lower and upper respiratory tract infections and children who had not visited a doctor may even so have suffered from a respiratory tract infection. However, since the outcome was measured after the breastfeeding period we do not expect such misclassification to be differential and to have influenced the effect of the duration or dose of breastfeeding. Also, our study did not have information on the number of episodes of infection. Li et al.[17] found a relation between two or more visits to the physician and breastfeeding duration and exclusivity.

Several long-term effects of breastfeeding on the offspring have been reported.[3–5, 15] Different mechanisms for the stimulation of the immune response by breastfeeding have been suggested, among others transfer of anti-idiotypic antibodies and lymphocytes.[6, 13] However, the mechanism by which breastfeeding might add to a long-term protection remain unclear.

In conclusion, this study showed that breastfeeding duration for 6 months or longer is associated with a reduced risk of lower respiratory tract infections in pre-school children. These findings are compatible with the hypothesis that the protective effect of the duration of breastfeeding for respiratory tract infections persist after infancy therefore supporting current WHO recommendations for breastfeeding for at least 6 months also in industrialized countries.

## Supporting information

S1 Fig Flowchart of the participants within the Generation R Study. (DOCX)

S1 Table Maternal and child characteristics (n = 5322). (DOCX)

S2 Table Association between breastfeeding duration and lower and upper respiratory tract infections up to age 4 years (ORIGINAL DATA). (DOCX)

S3 Table Association between breastfeeding dose and lower and upper respiratory tract infections up to age 4 years (ORIGINAL DATA). (DOCX)
