# Supplementary material for: Breastfeeding and the risk of respiratory tract infections after infancy: The Generation R Study
Source: PLoS One. 2017 Feb 23;12(2):e0172763. doi: 10.1371/journal.pone.0172763 (PMC5322970; doi:10.1371/journal.pone.0172763)
Supplement: S1 Fig — (DOCX) [file pone.0172763.s001.docx]

**S1 figure. Flowchart of the participants within the Generation R Study**

Mothers enrolled in the Generation R study (n=9778)

Provided consent for postnatal follow-up (n=7893)

Population of analysis
(n=5322)

Excluded: n=2015 due to no data available on RTI at age 2,3 or 4 years.
Excluded: n=556 random sample within siblings.

Excluded: n=1885 due to no consent for postnatal follow-up.

Data on upper respiratory tract infection

Age 2: n=4568
Age 3: n=4230
Age 4: n=4284

Data on lower respiratory tract infections

Age 2: n=4613
Age 3: n=4268
Age 4: n=4272

*Multiple imputed (see S1 table & table 2)*

*Multiple imputed (see S1 table & table 2)*
