# Supplementary material for: Breastfeeding and the risk of respiratory tract infections after infancy: The Generation R Study
Source: PLoS One. 2017 Feb 23;12(2):e0172763. doi: 10.1371/journal.pone.0172763 (PMC5322970; doi:10.1371/journal.pone.0172763)
Supplement: S1 Table — (DOCX) [file pone.0172763.s002.docx]

**S1 table. Maternal and child characteristics (n=5322)**

|  | | **Original data** | | **Multiple imputed** | |
| --- | --- | --- | --- | --- | --- |
| **Characteristics** | | **n** | **%** | **n** | **%** |
|  | |  |  |  |  |
| **Maternal characteristics** | |  |  |  |  |
|  | |  |  |  |  |
| Maternal age *Mean (SD)* | | 31.1 | 4.9 | 31.1 | 4.9 |
|  | *Missing* | 0 | 0 |  |  |
|  | |  |  |  |  |
| Educational level | |  |  |  |  |
|  | Low | 901 | 18 | 1004 | 19 |
|  | Mid | 2638 | 53 | 2799 | 52 |
|  | High | 1471 | 29 | 1519 | 29 |
|  | *Missing* | 312 | 6 |  |  |
|  | |  |  |  |  |
| Ethnicity | |  |  |  |  |
|  | Western | 3534 | 69 | 3652 | 69 |
|  | Non-Western | 1566 | 31 | 1670 | 31 |
|  | *Missing* | 222 | 4 |  |  |
|  | |  |  |  |  |
| Household income per month | |  |  |  |  |
|  | ≤ 2200 euro | 1556 | 37 | 2075 | 39 |
|  | >2200 euro | 2704 | 64 | 3247 | 61 |
|  | *Missing* | 1062 | 20 |  |  |
|  | |  |  |  |  |
| Marital status | |  |  |  |  |
|  | Married/ Living together | 4538 | 90 | 4788 | 90 |
|  | No partner | 497 | 10 | 534 | 10 |
|  | *Missing* | 287 | 5 |  |  |
|  | |  |  |  |  |
| Maternal BMI before pregnancy *Mean (SD)* | | 23.5 | 4.1 | 23.5 | 4.0 |
|  | *Missing* | 1325 | 25 |  |  |
|  | |  |  |  |  |
| Smoking during pregnancy | |  |  |  |  |
|  | Never | 3290 | 78 | 4114 | 77 |
|  | Smoked during pregnancy | 952 | 22 | 1208 | 23 |
|  | *Missing* | 1080 | 20 |  |  |
|  | |  |  |  |  |
| Smoking in presence of child | |  |  |  |  |
|  | Smoking mother | 543 | 16 | 912 | 17 |
|  | *Missing* | 1885 | 35 |  |  |
|  | Smoking in the home | 174 | 5 | 392 | 7 |
|  | *Missing* | 1884 | 35 |  |  |
|  | Smoking in other places | 448 | 13 | 802 | 15 |
|  | *Missing* | 1901 | 36 |  |  |
|  | |  |  |  |  |
| Alcohol use during pregnancy | |  |  |  |  |
|  | Never | 1803 | 42 | 2275 | 43 |
|  | Drank alcohol during pregnancy | 2466 | 58 | 3047 | 57 |
|  | *Missing* | 1053 | 20 |  |  |
|  | |  |  |  |  |
|  | |  |  |  |  |
|  | |  |  |  |  |
| **S1 table. Maternal and child characteristics (n=5322)** (Continued) | | | | | |
| Parental history of atopy | | 2459 | 49 | 2606 | 49 |
|  | *Missing* | 322 | 6 |  |  |
|  | |  |  |  |  |
| Multiparity | | 2111 | 41 | 2195 | 41 |
|  | *Missing* | 166 | 3 |  |  |
|  | |  |  |  |  |
| Caesarean section | | 660 | 14 | 733 | 14 |
|  | *Missing* | 538 | 10 |  |  |
|  | |  |  |  |  |
| Breastfeeding | |  |  |  |  |
|  | Never | 398 | 9 | 893 | 17 |
|  | < 3 months | 1466 | 35 | 1602 | 30 |
|  | 3-6 months | 860 | 20 | 1093 | 21 |
|  | ≥ 6 months | 1515 | 36 | 1734 | 32 |
|  | *Missing* | 1083 | 20 |  |  |
|  | |  |  |  |  |
| Breastfeeding | |  |  |  |  |
|  | Never | 406 | 10 | 862 | 16 |
|  | Partially until 4 months | 2647 | 64 | 2870 | 54 |
|  | Predominantly until 4 months | 1056 | 26 | 1590 | 30 |
|  | *Missing* | 1213 | 23 |  |  |
|  | |  |  |  |  |
| **Child characteristics** | |  |  |  |  |
|  | |  |  |  |  |
| Male | | 2667 | 50 | 2667 | 50 |
|  | *Missing* | 0 | 0 |  |  |
|  | | | | | |
| Preterm birth^1^ | | 268 | 5 | 272 | 5 |
|  | *Missing* | 25 | 1 |  |  |
|  | |  |  |  |  |
| Vitamin D supplementation age 6-12 months | | 1873 | 45 | 2489 | 71 |
|  | Missing | 1112 | 21 |  |  |
|  | |  |  |  |  |
| Day care attendance first 2 years | | 3140 | 95 | 4070 | 76 |
|  | *Missing* | 2013 | 38 |  |  |

^1^Preterm birth is defined as < 37 weeks.
