# Supplementary material for: Breastfeeding and the risk of respiratory tract infections after infancy: The Generation R Study
Source: PLoS One. 2017 Feb 23;12(2):e0172763. doi: 10.1371/journal.pone.0172763 (PMC5322970; doi:10.1371/journal.pone.0172763)
Supplement: S2 Table — (DOCX) [file pone.0172763.s003.docx]

**S2 table. Association between breastfeeding duration and lower and upper respiratory tract infections up to age 4 years** (ORIGINAL DATA)

|  |  | **Lower respiratory tract infections** | | **Upper respiratory tract infections** | |
| --- | --- | --- | --- | --- | --- |
| **Breastfeeding** |  | Univariate model | Multivariable model 1 | Univariate model | Multivariable model 1 |
|  | n (%) | OR (95 % CI) | aOR (95 % CI) ^a^ | OR (95 % CI) | aOR (95 % CI) ^a^ |
| Never | 398 (9%) | *Reference* | *Reference* | *Reference* | *Reference* |
| < 3 months | 1466 (35%) | **0.73 (0.55-0.96)** | 0.71 (0.41-1.21) | 0.97 (0.81-1.15) | 0.79 (0.56-1.14) |
| 3-6 months | 860 (20%) | **0.68 (0.50-0.92)** | 0.59 (0.34-1.04) | 0.87 (0.73-1.05) | 0.95 (0.66-1.37) |
| ≥ 6 months | 1515 (36%) | **0.59 (0.45-0.79)** | **0.56 (0.32-0.99)** | 0.86 (0.72-1.02) | 0.91 (0.63-1.31) |

OR: Odds Ratio; 95% confidence interval. OR’s are compared to never-breastfed.
^a^ Adjusted for caesarean section, maternal age, marital status, maternal ethnicity, maternal educational level, household income per month, maternal BMI before pregnancy, smoke exposure child, alcohol use during pregnancy, gender child, vitamin D supplementation age 6-12 months, day-care attendance in the first two years of life, gestational age at birth, parity and parental history of atopy.
